# Abnormal ferritin levels predict development of poor outcomes in cirrhotic outpatients: a cohort study

**DOI:** 10.1186/s12876-021-01669-w

**Published:** 2021-03-02

**Authors:** David Tornai, Peter Antal-Szalmas, Tamas Tornai, Maria Papp, Istvan Tornai, Nora Sipeki, Tamas Janka, Boglarka Balogh, Zsuzsanna Vitalis

**Affiliations:** 1 Department of Laboratory Medicine, Faculty of Medicine, University of Debrecen, 98 Nagyerdei krt., 4032 Debrecen, Hungary; 2 Kálmán Laki Doctoral School of Biomedical and Clinical Sciences, Faculty of Medicine, University of Debrecen, Debrecen, Hungary; 3 Division of Gastroenterology, Department of Internal Medicine, Faculty of Medicine, University of Debrecen, Debrecen, Hungary

**Keywords:** Ferritin, Cirrhosis, Outpatients, Long-term prognosis, Decompensation, Bacterial infection, Mortality

## Abstract

**Background:**

Both iron overload and iron deficient anemia can associate with cirrhosis. At the same time, inflammation might be continuously present in cirrhotic patients due to bacterial translocation and patients’ susceptibility to infections. Ferritin is a sensitive and widely available marker of iron homeostasis, in addition it acts as an acute phase protein. Therefore, we evaluated the prognostic potential of serum ferritin in the long-term follow-up of cirrhotic outpatients.

**Methods:**

A cohort of 244 cirrhotic outpatients was recruited and followed for 2 years. We measured their serum ferritin levels in our routine laboratory unit at enrolment and investigated its association with clinical outcomes.

**Results:**

Ferritin serum level was higher in males and older patients than in females (median: 152.6 vs. 75 μg/L, *p* < 0.001) or younger individuals (median: 142.9 vs. 67.9 μg/L, *p* = 0.002). Patients who previously survived variceal bleeding had lower ferritin levels (median: 43.1 vs. 146.6 μg/L, *p* < 0.001). In multivariate regression models, including laboratory and clinical factors, lower (< 40 μg/L) ferritin concentration was associated with the development of decompensated clinical stage in patients with previously compensated cirrhosis (sHR: 3.762, CI 1.616–8.760, *p* = 0.002), while higher (> 310 μg/L) circulating ferritin levels were associated with increased risks of bacterial infections in decompensated patients (sHR: 2.335, CI 1.193–4.568, *p* = 0.013) and mortality in the whole population (HR: 2.143, CI 1.174–3.910, *p* = 0.013).

**Conclusion:**

We demonstrated usefulness of serum ferritin as a prognostic biomarker in cirrhosis, pointing out that both low and high concentrations need attention in these patients.

## Introduction

Different metabolic and hematological abnormalities often associate with different chronic liver diseases. Both iron overload and iron deficient anemia are reported in patients with liver cirrhosis (LC) [1, 2].

Iron overload in LC seems to be due, at least in part, to reduced hepcidin levels caused by decreased synthetic capacity of hepatocytes. The role of hepcidin is to inhibit iron uptake by ferroportin in the basal membrane of enterocytes. Therefore, decreased hepcidin concentration leads to increased iron uptake [3, 4]. Additionally, infection/inflammation also decreases hepcidin levels [4]. Endotoxemia, driven by bacterial translocation, is common in LC and maintains a chronic inflammatory state in these patients. At the same time, since cirrhosis is an immune suppressed condition, especially in the later decompensated state of the disease, infections appear more frequently and show a more severe course in cirrhotic patients than in the healthy population [5, 6]. Thus, in LC, hepcidin synthesis can be suppressed both chronically (deteriorating liver function and chronic inflammation) and acutely (infections), leading to increased iron absorption and elevated free iron levels. Iron, loaded in the liver cells or macrophages, was shown to be toxic, potentiates oxidative stress and induces peroxidation, resulting in further liver cell damage [7–9].

On the other hand, acute and chronic bleedings are common in cirrhotic patients. Portal hypertension and instable hemostasis increase the risk of variceal bleeding, portal hypertensive gastropathy, gastric antral vascular ectasia and peptic ulcer bleeding. Malabsorption may also be present in cirrhotic individuals contributing to reduced iron storages [2]. Iron deficiency in return was shown to contribute to lipid, glucose and nutrient metabolic dysfunction, as well as induction of cell apoptosis [10, 11].

Serum ferritin level is a sensitive marker of iron homeostasis in the healthy population; reduced iron levels are associated with low ferritin, while iron overload results in higher serum ferritin concentrations, by modulating iron-response elements and their binding proteins. However, in cirrhosis, the interpretation of ferritin level is more complicated since ferritin is an acute phase protein. Therefore, its concentration also increases due to inflammation [2, 12]. As discussed above, both inflammation and iron overload might be present in LC. Accordingly, elevated hepatic and serum ferritin levels are consistently reported in chronic liver diseases [13–15].

Ferritin is primarily an intracellular protein, which stores up to 4500 iron atoms per molecule in a safe but bioavailable form. It is composed of two types of subunits, termed H and L, the ratio of which varies by organ and cell type [16]. Ferritin secretion has been demonstrated via non-canonical pathways in macrophages, hepatocytes and Kupffer cells of the liver and it was shown to be mostly composed of the L-subunit [17–19]. However, the function of circulating ferritin is still not fully understood.

Despite our tendency to consider serum ferritin as iron-poor, it usually incorporates hundreds of iron atoms, which is a substantial amount compared to the two atoms delivered by transferrin. Therefore, studies have proposed that serum ferritin might also have a role in iron transport, especially at times of increased iron demand [20, 21]. Ferritin is also secreted in response to inflammation and has been reported to have anti-inflammatory properties. Serum ferritin can bind to high molecular weight kininogen, preventing its cleavage and leading to the reduction in bradykinin release [22].

Considering all of its functions, it seems tempting to hypothesize that ferritin can mark several pathological processes or even play a substantial role in the progression of LC. Ferritin measurement is a widely available and inexpensive method making it a potentially easy-to-use prognostic marker. Previous studies found an association between high ferritin levels and increased risk of short-term mortality in cirrhotic patients with acute decompensation (AD) [23–25], but the long-term prognostic value of ferritin has not been reported. Therefore, we investigated serum ferritin levels in clinically stable outpatients with cirrhosis and evaluated its association with the development of decompensated clinical stage, bacterial infections (BI) and mortality.

## Patients and methods

### Patient population

We recruited adult patients with established diagnoses of cirrhosis of different etiologies from the outpatient clinic at our gastroenterology tertiary care referral center during regular or unscheduled follow-up visits. In total 244 well-characterized cirrhotic patients with 2 years of clinical follow-up were included between May 1, 2006 and October 31, 2011. The diagnosis of cirrhosis was based on the combination of clinical (cutaneous signs, liver stiffness by palpation, ascites, presence of collateral varices, and/or predisposing factors, such as alcohol abuse, metabolic syndrome, viral hepatitis and autoimmune liver disease), biochemical (elevated bilirubin and coagulation parameters, decreased albumin, cholesterol and cholinesterase level and platelet number), imaging (unevenness of the liver surface, increased liver density, inhomogeneity, splenomegaly, ascites by ultrasonography or computer tomography), elastography results (after it became accessible in 2009 at our Institute) and when available, histological (n = 62) data. Additionally, cirrhosis of 13 patients was recognized by surgeons at time of abdominal surgery. Clinical characteristics of patients at enrolment are presented in Table 1.

**Table 1:** Epidemiological, clinical and laboratory characteristics of patients with cirrhosis

		Patients		
		All n = 244	Compensated n = 101	Decompensated n = 143
Age^#^		56 (49–65)	54 (49–64)	57 (51–65)
Sex (m/f)		124/120	47/54	77/66
Alcoholic etiology*		137 (56.1%)	36 (35.6%)	101 (70.6%)
Child-	A	153 (62.7%)	97 (96.0%)	56 (39.2%)
Pugh	B	80 (32.8%)	4 (4.0%)	76 (53.1%)
Stage*	C	11 (4.5%)	0 (0.0%)	11 (7.7%)
MELD score^#^		11 (8–14)	9 (7–12)	12 (9–14)
Bilirubin (μmol/L)^#^		26 (15–42)	18 (13–30)	30 (19–51)
Albumin (g/L)^#^		38 (33–42)	42 (37–45)	36 (31–40)
Creatinine (μmol/L)^#^		67 (54–83.5)	62 (52–76)	70 (55–88)
INR^#^		1.17 (1.09–1.31)	1.11 (1.05–1.21)	1.22 (1.12–1.36)
WBC (10^3^/ml)		5.26 (4.19–7.14)	5.55 (4.25–7.85)	5.24 (4.05–6.89)
CRP (mg/L)^#^		3.84 (1.19–7.94)	1.41 (0.50–3.95)	5.67 (2.58–9.87)
Anemia*^o^		98 (40.2%)	23 (22.8%)	75 (52.4%)
Previous VB*		59 (24.2%)	0 (0.0%)	59 (41.3%)
Ascites*		88 (36.1%)	0 (0.0%)	88 (61.5%)
Comorbidity*		127 (52%)	47 (46.5%)	80 (55.9%)
HCC*		26 (10.7%)	12 (11.9%)	14 (9.8%)
Ferritin (μg/L)^#^		121.2 (39.7–310.8)	133.7 (49.2–370.1)	103.4 (35.8–271.9)
Ferritin (L/M/H)		61/122/61	22/48/31	39/74/30

*m/f* male/female, *MELD* model for end stage liver disease, *INR* international normalized ratio, *WBC* white blood cells, *CRP* C-reactive protein, *VB* variceal bleeding, *HCC* hepatocellular carcinoma, *L/M/H* low/medium/high

^#^Median (interquartile range); *n (%); ^o^determined by low hemoglobin and/or hematocrit levels

**Fig. 1:**
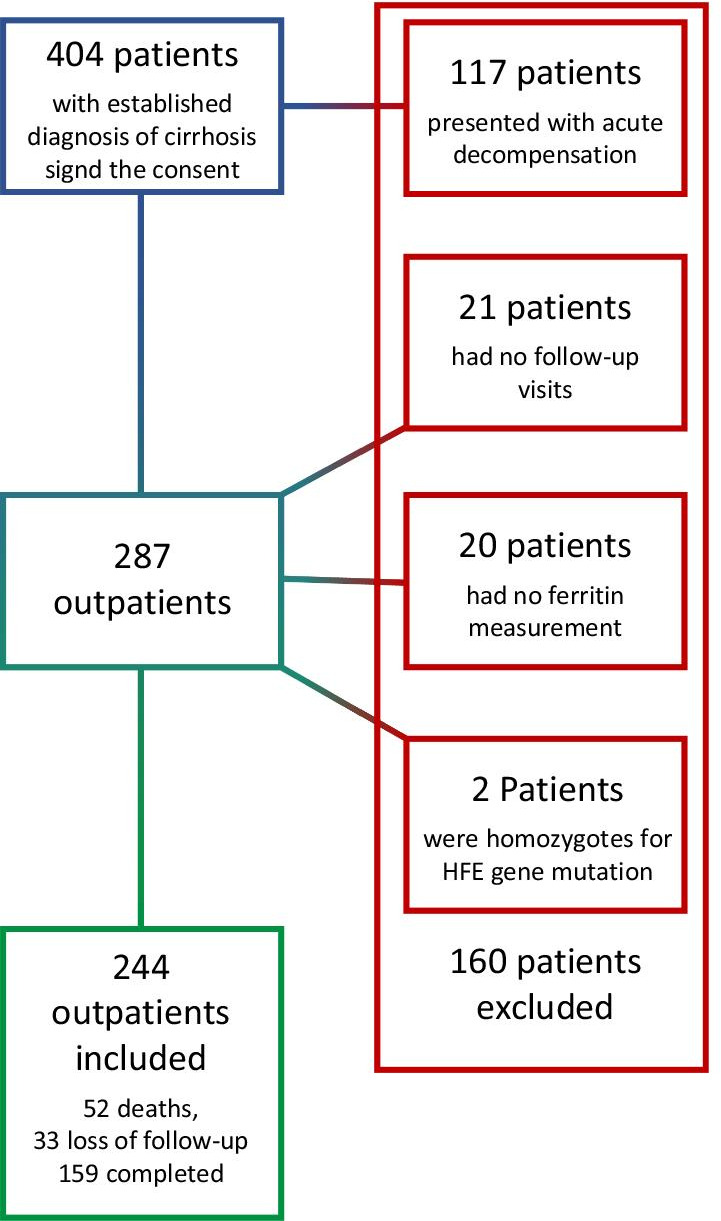
Exclusion process of patients with cirrhosis

The exclusion criteria were (1) the patient or his/her legal guardian declined to participate in the study and did not sign the informed consent, (2) the patient was sent only for a specialist consultation, but was regularly followed-up elsewhere, (3) a positive hemochromatosis test or (4) the presence of AD. AD was defined by acute development of large ascites (grade II/III), acute hepatic encephalopathy (HE), acute variceal bleeding and/or presence of BI warranted hospital admission [6] (Fig. 1).

Blood samples, routine laboratory data and detailed clinical phenotype were captured at inclusion. Clinical data were determined by an in-depth review of the patients’ medical records using a structured interview. Medical records that documented age at diagnosis, etiology, presence of hepatocellular carcinoma, esophageal varices, extrahepatic co-morbidities (myocardial infarction, congestive heart failure, peripheral arterial disease, cerebrovascular disease, chronic pulmonary disease, chronic renal failure, diabetes mellitus, extrahepatic malignant disease), history of previous AD episode(s), and cirrhosis-related medication were retrospectively analyzed for the period prior to the observational follow-up study. At enrolment, disease severity (assessed by liver-oriented scores: Child–Pugh and MELD) and the clinical stage of the diseases as described by D’Amico et al. [6] were determined. We used this classification to divide our patients into compensated and decompensated groups (i.e., patients with or without previous decompensation episodes) when assessing predictive value of ferritin level.

To evaluate the association between serum ferritin levels and other parameters of hematopoiesis, we categorized patients into anemic and non-anemic groups according to their hemoglobin and hematocrit values. In our routine diagnostic laboratory, the normal ranges of hemoglobin and hematocrit are 115–150 g/L and 0.35–0.47 for females and 130–165 g/L and 0.39–0.50 for males, respectively. Patients were categorized into the anemic group if at least one of these parameters was below the lower limit of the reference range.

During the observational period, the attending gastroenterologist recorded date and type of the developed adverse events (AD, BI, mortality) according to established clinical guidelines as described previously [26]. Diagnosis of BI development was based on compatible clinical symptoms, imaging findings (abdominal ultrasound and chest X-ray) and laboratory data. Laboratory data consisted of elevated leucocyte count either absolute (> 10.8 G/L) or relative (double of count at former visits) in patients with leukopenia with an elevated neutrophil rate (> 76%) and increased serum levels of CRP (> 10.0 mg/L) and/or PCT (> 0.15 μg/L) and results of urine analysis (sediment). When ascites was present, the result of diagnostic tap (neutrophil count and ascites culture) was considered. Based on the results of this procedure, cultures from specific sites (sputum, urine, wound discharge, etc.) were obtained according to location of infection. In sepsis, or when the infection location could not be clearly identified, blood cultures were obtained. The follow-up period lasted 2 years, or until death or the loss of follow-up. Collected data were transferred and stored in a database. At the end of the study period, all clinical data were extracted for further analysis.

The research was performed in accordance with the Declaration of Helsinki and the study protocol was approved by the Regional and Institutional Research Ethics Committee of the University of Debrecen and the National Scientific and Research Ethics Committee (DEOEC-RKEB/IKEB 5306-9/2011, 3885/2012/EKU [60/PI/2012]).

### Serological analysis

Blood samples were obtained at enrolment. Ferritin level was measured by a two-site chemiluminescence immunoassay (Roche, Basel, Switzerland) using the Cobas e602 analyzer (Roche) in our routine laboratory unit. The lower assay sensitivity limit was 0.5 μg/L. As there is no consensus on pathologic ferritin levels in cirrhotic patients, in our study, low (< 40 μg/L) and high (> 310 μg/L) ferritin levels were determined arbitrarily corresponding to the patients’ 25th and 75th serum level percentile (i.e., 1st and 4th quartile [Q1 and Q4]).

### Statistical analysis

Variables were tested for normality using Shapiro Wilk’s W test. Continuous variables were summarized as median and interquartile range (IQR [lowest 25%–highest 25%]) and compared with Mann–Whitney U test for two groups or Kruskal–Wallis H test with Dunn’s multiple comparison post hoc analysis for three or more groups. Spearman’s nonparametric rank correlation test was used to determine correlations. Kaplan–Meier analysis was used to calculate the cumulative probability of mortality. Differences in observed probabilities were assessed with the log-rank test. The association between categorical clinical variables or serum ferritin level and mortality during follow-up was assessed with univariate Cox-regression analysis. For adverse outcomes with competing events, we used the Aalen-Johansen estimator, a modified version of the Kaplan–Meier analysis, that takes competing outcomes into account, to plot the cumulative incidence function. The association between these events, clinical factors and serum ferritin levels was calculated by univariate Fine-Gray proportional hazard regression. Multivariate analyses were performed with backward elimination procedure and likelihood ratio test to identify independent predictors. Associations are provided as hazard ratio (HR) or sub-distribution hazard ratio (sHR) with 95% confidence intervals (CI). For the Fine-Gray test, we utilized R 3.5.1 supplemented with the EZR package, R commander and RcmdrPlugin.EZR plugin. For all other statistical analyses and graphical presentation, the SPSS 25.0 (SPSS, Chicago, IL), and GraphPad Prism 8.4 (San Diego, CA) programs were used. A 2-sided probability value of < 0.05 was considered to be statistically significant.

## Results

### Association of ferritin with clinical and laboratory parameters

**Fig. 2:**
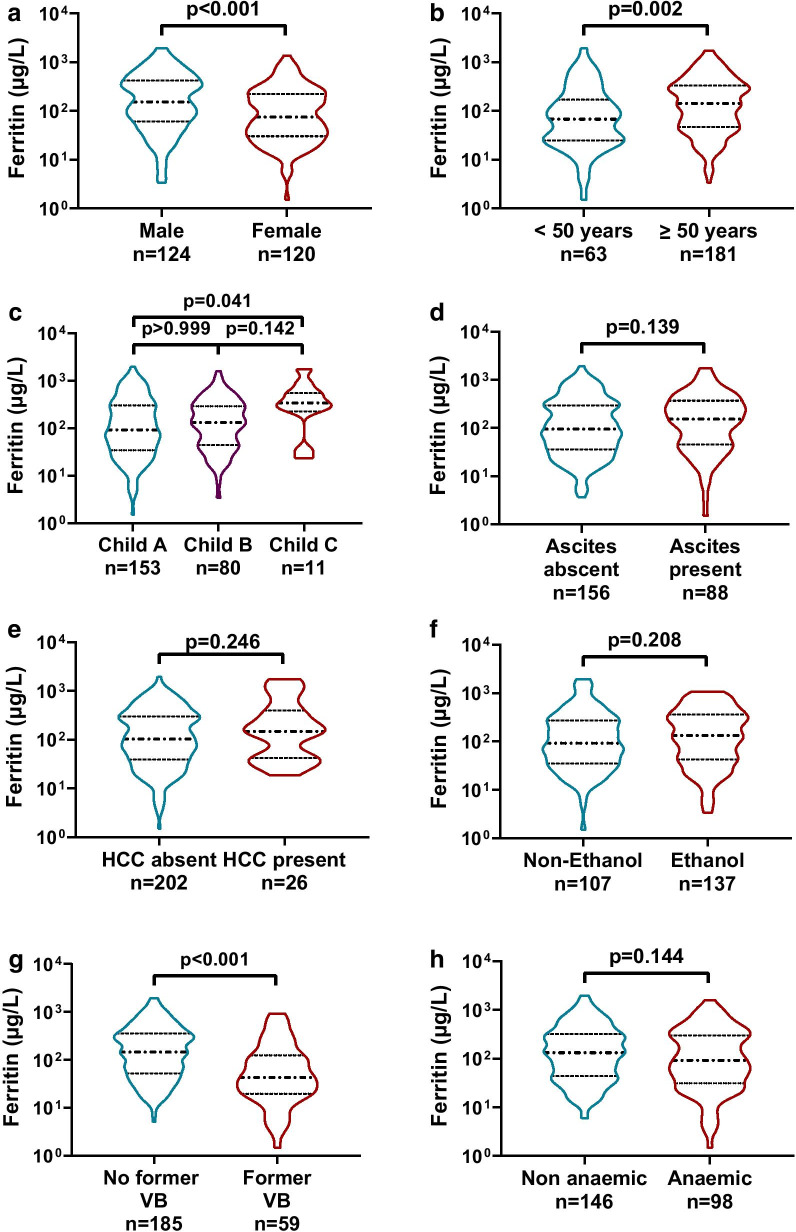
Serum ferritin levels according to different patient characteristics. Ferritin levels were higher in male (**a**) and older (**b**) compared to female or younger patients. According to patients’ severity, Child–Pugh C stadium was associated with increased ferritin levels only compared to Child–Pugh A stadium (**c**). There was no difference between groups by the presence of ascites (**d**), hepatocellular carcinoma ([HCC] **e**) or alcoholic etiology (**f**). Patients with a history of variceal bleeding (VB) had significantly lower serum ferritin levels (**G**), while there was no difference detected between patients with or without anemia (**h**) determined by low hemoglobin and/or hematocrit levels

Serum levels of ferritin ranged from 1.5 to 1954.6 μg/L in our cirrhotic patient cohort. Increased ferritin levels were found in males compared to females (median [IQR]: 152.6 [60.9–420.4] vs. 75.0 [30.3–223.5] μg/L, *p* < 0.001; Fig. 2a), and in ≥ 50 years old patients compared to younger ones (median [IQR]: 142.9 [47.0–333.0] vs. 67.9 [24.8–170.8] μg/L, *p* = 0.002; Fig. 2b). When we categorized our patients according to disease severity as described by Child–Pugh stages (median [IQR]: A: 92.0 [34.2–303.4]; B: 131.1 [44.2–289.0]; C: 340.1 [227.1–548.4] μg/L) we only observed statistically significant difference between the A and C groups (*p* = 0.041; Fig. 2c). Furthermore, we didn’t detect significant differences in serum ferritin levels between patients with or without ascites (median [IQR]: 153.4 [45.4–370.7] vs. 95.0 [36.3–297.0] μg/L, *p* = 0.139; Fig. 2d), in the absence or presence of HCC (median [IQR]: 103.8 [39.3–301.4] vs. 149.1 [42.7–399.6] μg/L, *p* = 0.246, Fig. 2e), and with alcoholic or non-alcoholic etiology (median [IQR]: 133.2 [42.7–363.1] vs. 92.0 [35.0–271.9] μg/L, *p* = 0.208, Fig. 2f).

Importantly, ferritin levels were significantly lower in patients who had a prior variceal bleeding episode compared to those who did not have such an event (median [IQR]: 43.1 [19.8–125.6] vs. 146.6 [52.7–358.3] μg/L, *p* < 0.001, Fig. 2g). However, when we divided our patients according to the presence or absence of anemia as described in the Methods section, we did not find significant differences between the two groups (median [IQR]: 92.5 [31.2–301.4] vs. 132.4 [43.8–320.0] μg/L, respectively; *p* = 0.144, Fig. 2h).

Next, we investigated the association between serum ferritin level and other laboratory parameters (i.e., continuous variables). While ferritin levels correlated with markers of hematopoiesis and iron homeostasis in both compensated and decompensated patients, the indicators of inflammation (CRP and white blood cell counts) showed correlation with ferritin levels only in patients with previous decompensation. Similarly, disease severity measures (Child–Pugh score and MELD) correlated with ferritin only in the decompensated group. Non-parametric correlations are summarized in Table 2.

**Table 2:** Spearman correlation of ferritin levels with laboratory parameters

	All patients		Compensated		Decompensated	
	Spearman r	*p* value	Spearman r	*p* value	Spearman r	*p* value
MELD score	0.107	0.104	− 0.018	0.868	0.287	**0.001**
Child–Pugh score	0.144	**0.027**	0.117	0.263	0.336	**< 0.001**
Albumin	− 0.018	0.777	0.048	0.635	− 0.136	0.107
Bilirubin	0.125	0.053	0.080	0.426	0.247	**0.003**
Creatinine	0.175	**0.007**	0.201	0.051	0.186	**0.027**
INR	− 0.044	0.503	− 0.167	0.109	0.124	0.140
AST	0.236	**< 0.001**	0.173	0.088	0.271	**0.002**
ALT	0.228	**< 0.001**	0.203	**0.045**	0.244	**0.005**
γGT	0.225	**< 0.001**	0.328	**0.001**	0.133	0.131
CRP	0.127	0.062	0.071	0.522	0.229	**0.008**
WBC	0.174	**0.011**	0.060	0.599	0.256	**0.003**
Neutrophil count	0.108	0.120	0.019	0.868	0.182	**0.040**
Lymphocyte count	0.166	**0.017**	0.097	0.391	0.212	**0.016**
sCD163	0.170	**0.011**	0.223	**0.033**	0.124	0.154
IgG	− 0.179	**0.006**	− 0.246	**0.014**	− 0.132	0.122
IgA	0.104	0.112	0.066	0.518	0.193	**0.023**
Hemoglobin	0.313	**< 0.001**	0.264	**0.008**	0.370	**< 0.001**
Hematocrit	0.300	**< 0.001**	0.290	**0.004**	0.327	**< 0.001**
MCH	0.510	**< 0.001**	0.360	**< 0.001**	0.634	**< 0.001**
MCV	0.487	**< 0.001**	0.312	**0.002**	0.629	**< 0.001**
Age	0.149	**0.020**	0.175	0.080	0.148	0.078

Values in bold indicate statistically significant tests

*ALP* alkaline phosphatase, *ALT* alanine aminotransferase, *AST* aspartate aminotransferase, *CRP* C-reactive protein, *γGT* γ-glutamyl-transferase, *INR* international normalized ratio, *MCH* mean corpuscular hemoglobin, *MCV* mean corpuscular volume, *MELD* model for end stage liver disease, *WBC*, white blood cells

### Low serum ferritin level is associated with increased 2-year incidence of developing a decompensated clinical stage

Of 244 patients, 143 (58.6%) experienced at least one form of decompensation episodes (ascites development, variceal bleeding, HE) prior to enrolment (decompensated patients), and 101 (41.4%) had medical histories free from these events (compensated patients).

In patients with compensated cirrhosis, we investigated the impact of serum ferritin levels on the development of decompensated clinical stage during the 2-year follow-up period. We registered such events in 20 of our previously compensated patients (19.8%), of which 4 were associated with BI. As the first AD, 15 patients had ascites, 8 HE and 6 variceal bleeding. In 3 cases, all three events developed simultaneously, in 2 of them, ascites occurred in combination with HE, while in one case, ascites and variceal bleeding appeared together.

**Fig. 3:**
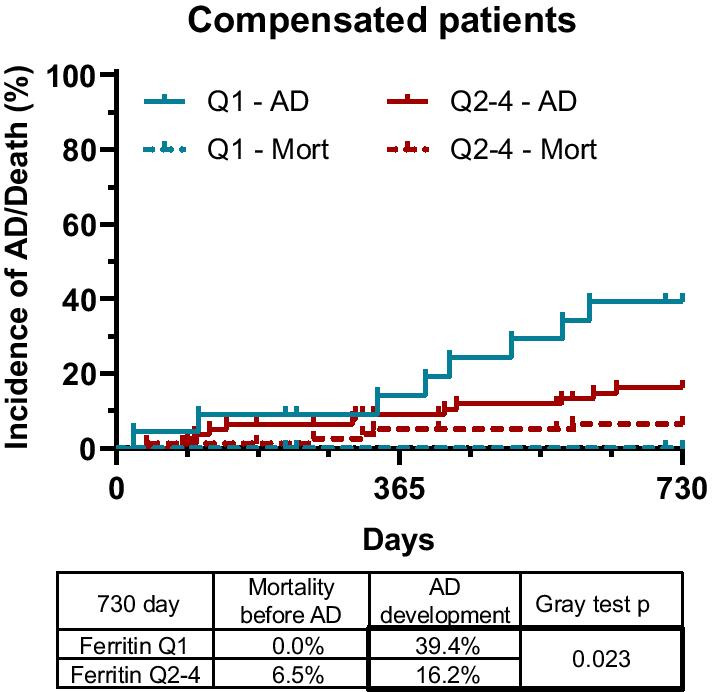
Low serum ferritin levels are associated with 2-year development of decompensated clinical stage in compensated cirrhotic patients. Aalan–Johansen estimator taking mortality into account as a competing event indicates that low (Q1) serum ferritin levels were associated with an increased incidence of first acute decompensation (AD) development in previously compensated patients

We summarized these episodes and plotted Aalan–Johansen graphs. Low (Q1) ferritin levels were associated with an increased incidence of first appearing AD events (39.4%) compared to higher values (Q2–4; 16.2% *p* = 0.023; Fig. 3) during the 2-year follow-up.

In univariate Fine-Gray proportional hazard regression analysis, low (Q1) serum ferritin concentration was found to be significantly associated with the development of first AD events (sHR: 2.782, CI 1.156–6.696, *p* = 0.022) besides 5-point increase in MELD score, presence of comorbidities, HCC, higher than 10 mg/L CRP serum level and anemia (Table 3). In the subsequent multivariate Fine-Gray proportional hazard regression model with backward elimination, 5-point increase in MELD score, presence of HCC, anemia and low (Q1) serum ferritin concentration (sHR: 3.762, CI 1.616–8.760, *p* = 0.002) were independent risk factors of first AD development (Table 3).

**Table 3:** 2-year development of decompensated clinical stage in previously compensated cirrhotic patients

Compensated patients						
2-year development of first AD event	Univariate Fine-Gray regression			Multivariate Fine-Gray regression		
	sHR	CI (95%)	*p* value	sHR	CI (95%)	*p* value
Age (≥ 50)	1.268	0.461–3.485	0.650			
Sex (female)	0.543	0.225–1.315	0.180			
Alcoholic etiology	1.968	0.823–4.706	0.130			
MELD (5-point increase)	**2.592**	**1.255–5.336**	**0.010**	**2.738**	**1.134–6.607**	**0.025**
Child–Pugh B^a^	1.394	0.158–12.260	0.760			
Presence of comorbidity	2.421	0.965–6.076	0.060	1.157	0.454–2.949	0.760
Presence of HCC	**2.862**	**1.091–7.509**	**0.033**	**3.712**	**1.386–9.946**	**0.009**
CRP (≥ 10)	**3.211**	**1.156–8.924**	**0.025**	2.821	0.842–9.453	0.093
Anemia	**4.113**	**1.731–9.772**	**0.001**	**3.193**	**1.259–8.101**	**0.015**
Low Ferritin	**2.782**	**1.156–6.696**	**0.022**	**3.762**	**1.616–8.760**	**0.002**

Values in bold indicate statistically significant tests

*MELD* model for end stage liver disease, *HCC* hepatocellular carcinoma, *CRP* C-reactive protein, *sHR* sub-distribution hazard ratio, *CI* confidence interval

^a^There was no Child-C stage patient in compensated group

### High serum ferritin level is associated with increased 2-year development of BI

**Fig. 4:**
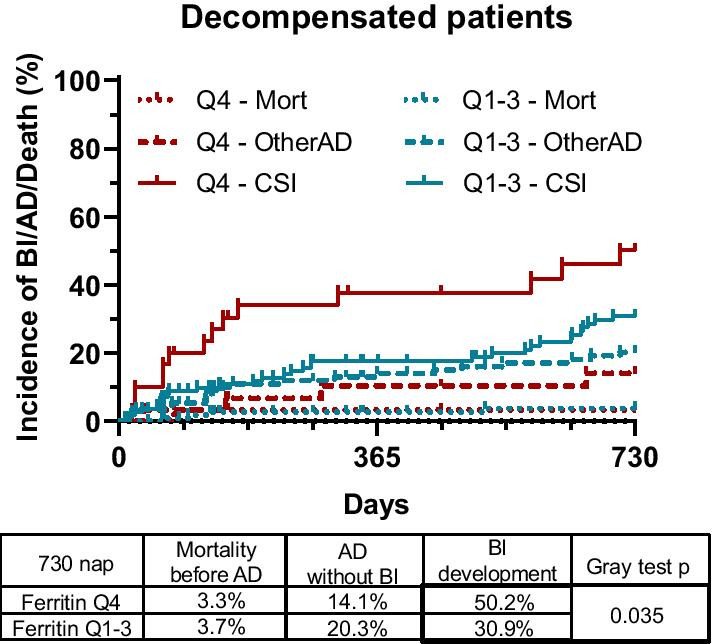
High serum ferritin levels are associated with 2-year development of bacterial infection (BI) in decompensated clinical stage cirrhotic patients. Aalan–Johansen estimator taking both AD episodes without BI and mortality into account as competing events indicates that in decompensated patients, high (Q4) ferritin levels were associated with an increased incidence of clinically significant bacterial infection (BI) development (**b**)

In decompensated cirrhotic patients, we evaluated the incidence of BI development, as it is the most frequent cause of morbidity and mortality in this considerably immunocompromised state of the disease. Seventy of these patients developed at least one AD episode during follow-up, and 45 of these cases were attributed to BIs. Therefore, in this population, we took AD events both with and without BI as well as mortality into account when plotting Aalan–Johansen graphs. Corresponding to an increased inflammatory state, in this population, high (Q4) ferritin levels were associated with an increased incidence of BI development (50.2%) compared to lower values (Q1–3; 30.9%, *p* = 0.035; Fig. 4) during the two-year follow-up.

In univariate Fine-Gray proportional hazard regression analysis, age of 50 or more, female sex of the patients, presence of ascites, previous BI episode, > 10 mg/L CRP serum level and high (Q4) serum ferritin concentration (sHR: 1.991, CI 1.054–3.758, *p* = 0.034) were associated with the development of BI (Table 4). Multivariate Fine-Gray regression model indicated that female sex of the patients, presence of ascites, previous BI episode and high (Q4) serum ferritin concentration (sHR: 2.335, CI 1.193–4.568, *p* = 0.013) were independent risk factors of 2-year BI development (Table 4).

**Table 4:** 2-year development of clinically significant bacterial infection (BI) in decompensated cirrhotic patients

Decompensated patients							
2-year BI development		Univariate Fine-Gray regression			Multivariate Fine-Gray regression		
		sHR	CI (95%)	*p* value	sHR	CI (95%)	*p* value
Age (≥ 50)		**3.297**	**1.206–9.016**	**0.020**	2.362	0.846–6.597	0.100
Sex (female)		**2.099**	**1.150–3.832**	**0.016**	**2.347**	**1.284–4.288**	**0.006**
Alcoholic etiology		1.568	0.759–3.243	0.220			
MELD (5-point increase)		1.239	0.811–1.895	0.320			
Child–Pugh	A	**Reference**					
	B	1.398	0.913–2.141	0.120			
	C	2.036	0.667–6.220	0.212			
Presence of comorbidity		1.241	0.678–2.272	0.480			
Presence of HCC		1.366	0.516–3.614	0.530			
Presence of ascites		**2.105**	**1.099–4.031**	**0.025**	**1.902**	**1.013–3.571**	**0.045**
Previous VB		0.922	0.520–1.636	0.780			
Previous BI		**2.348**	**1.279–4.310**	**0.006**	**2.560**	**1.374–4.768**	**0.003**
CRP (≥ 10)		**2.068**	**1.089–3.929**	**0.026**	1.577	0.767–3.243	0.220
Anemia		1.316	0.732–2.365	0.360			
High Ferritin		**1.991**	**1.054–3.758**	**0.034**	**2.335**	**1.193–4.568**	**0.013**

Values in bold indicate statistically significant tests

*MELD* model for end stage liver disease, *HCC* hepatocellular carcinoma, *VB* variceal bleeding, *BI* bacterial infection, *CRP* C-reactive protein, *sHR* sub-distribution hazard ratio, *CI* confidence interva

### High serum ferritin level is associated with increased 2-year mortality

**Fig. 5:**
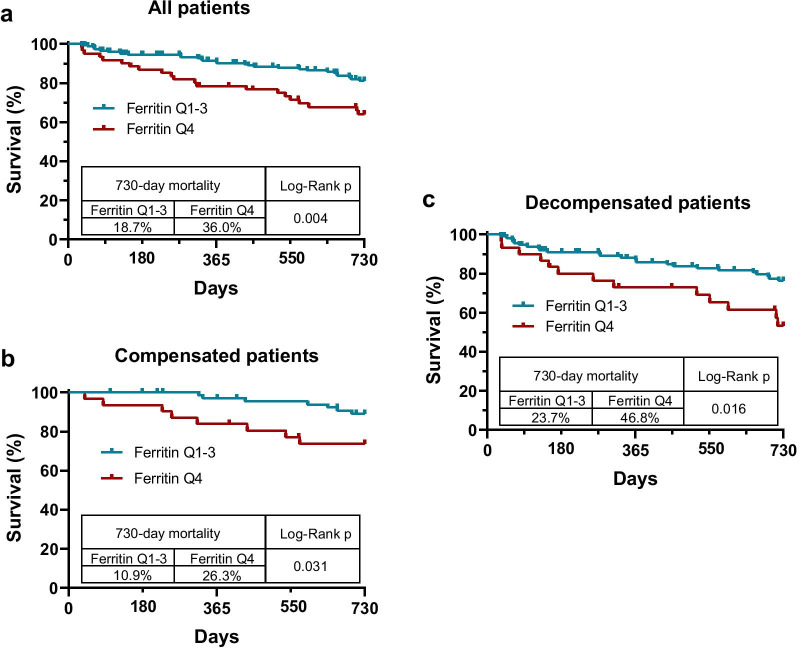
High serum ferritin levels are associated with 2-year mortality. Kaplan–Meier curves demonstrate higher incidence of mortality associated with high (Q4) ferritin levels in the whole cohort (**a**) and in the separate compensated (**b**) and decompensated (**c**) patient groups

We evaluated the potential impact of serum ferritin level on mortality in the whole cohort as well as in both compensated and decompensated patient populations. High ferritin levels were found to be associated with increased mortality rate in all these groups assessed with Kaplan–Meier analysis (Fig. 5a–c).

**Fig. 6:**
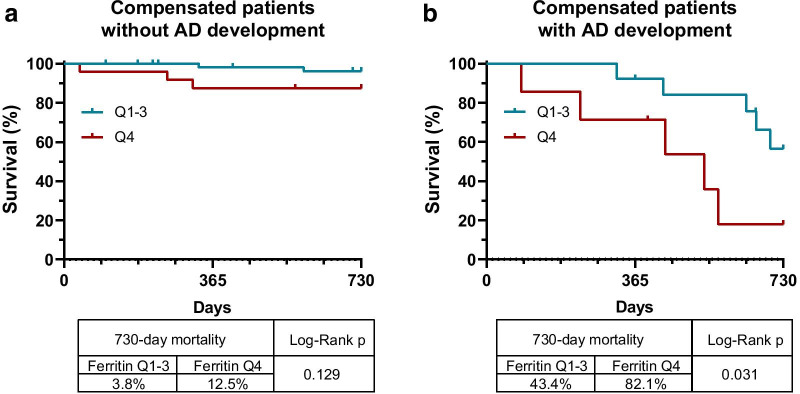
High serum ferritin levels are associated with 2-year mortality after first decompensation. Kaplan–Meier curves demonstrate higher incidence of mortality associated with high (Q4) ferritin levels in previously compensated patients who developed decompensated clinical stage during follow-up. In patients who remained compensated, only a trend was detected

We found the same association even when we divided compensated patients according to who did and did not develop decompensated disease stage during follow-up. In both subpopulations, mortality showed higher incidence in patients with high (Q4) ferritin levels. However, due to the low number of events in the group that remained compensated, this association reached statistical significance only in patients who developed decompensated clinical stage (Fig. 6).

Univariate Cox regression analyses also indicated association between high (Q4) ferritin levels and 2-year mortality in every group (all: HR: 2.209, CI 1.269–3.844, *p* = 0.005; compensated: HR: 2.903, CI 1.052–8.012, *p* = 0.040; decompensated: HR: 2.251, CI 1.146–4.424, *p* = 0.019). Relevant clinical and laboratory factors were also evaluated (Table 5).

**Table 5:** A summary and comparsions of typical examples for other recent underwater superoleophbic paper-based materials

A: All patients							
2-year mortality		Univariate Cox regression			Multivariate Cox regression		
		HR	CI (95%)	*p* value	HR	CI (95%)	*p* value
Age (≥ 50)		**4.341**	**1.565–12.040**	**0.005**	2.669	0.945–7.541	0.064
Sex (female)		0.98	0.569–1.688	0.941			
Alcoholic etiology		1.341	0.762–2.358	0.309			
MELD (5-point increase)		**2.029**	**1.482–2.776**	**< 0.001**	**1.758**	**1.201–2.572**	**0.004**
Child–Pugh	A	**Reference**					
	B	**2.936**	**1.167–5.223**	**< 0.001**	**2.617**	**1.408–4.861**	**0.002**
	C	**4.834**	**1.817–12.857**	**0.002**	1.950	0.625–6.079	0.250
Decompensated state		**2.021**	**1.109–3.683**	**0.022**	0.969	0.428–2.194	0.940
Presence of comorbidity		**2.244**	**1.245–4.046**	**0.007**	1.270	0.665–2.429	0.469
Presence of HCC		**5.642**	**3.064–10.388**	**< 0.001**	**8.827**	**4.423–17.618**	**< 0.001**
CRP (≥ 10)		**3.021**	**1.674–5.450**	**< 0.001**	1.567	0.799–3.076	0.191
Anemia		**1.969**	**1.141–3.400**	**0.015**	1.420	0.810–2.490	0.221
High Ferritin		**2.209**	**1.269–3.844**	**0.005**	**2.143**	**1.174–3.910**	**0.013**

B: Compensated patients						
2-year mortality	Univariate Cox regression			Multivariate Cox regression		
	HR	CI (95%)	*p* value	HR	CI (95%)	*p* value
Age (≥ 50)	2.886	0.651–12.791	0.163			
Sex (female)	0.583	0.207–1.638	0.306			
Alcoholic etiology	1.235	0.439–3.469	0.689			
MELD (5-point increase)	2.135	0.936–4.869	0.071	**2.595**	**1.088–6.188**	**0.032**
Child–Pugh B	0.047	0.000–5234	0.606			
Presence of comorbidity	1.947	0.692–5.476	0.207			
Presence of HCC	**4.691**	**1.593–13.809**	**0.005**	**9.211**	**2.854–29.731**	**< 0.001**
CRP (≥ 10)	1.789	0.404–7.929	0.444			
Anemia	2.506	0.890–7.057	0.082	1.333	0.424–4.184	0.623
High Ferritin	**2.903**	**1.052–8.012**	**0.040**	**4.367**	**1.466–13.009**	**0.008**

C: Decompensated patients							
2-year mortality		Univariate Cox regression			Multivariate Cox regression		
		HR	CI (95%)	*p* value	HR	CI (95%)	*p* value
Age (≥ 50)		**5.303**	**1.275–22.051**	**0.022**	2.608	0.602–11.308	0.200
Sex (female)		1.317	0.691–2.509	0.403			
Alcoholic etiology		0.964	0.466–1.993	0.921			
MELD (5-point increase)		**1.833**	**1.271–2.643**	**0.001**	**1.639**	**1.096–2.452**	**0.016**
Child–Pugh	A	**Reference**					
	B	**3.940**	**1.620–9.582**	**0.002**	**2.514**	**1.003–6.301**	**0.049**
	C	**6.024**	**1.831–19.815**	**0.003**	1.888	0.493–7.227	0.353
Presence of comorbidity		**2.196**	**1.063–4.538**	**0.034**	1.479	0.657–3.327	0.344
Presence of HCC		**8.225**	**3.810–17.756**	**< 0.001**	**8.058**	**3.461–18.759**	**< 0.001**
Presence of ascites		**3.461**	**1.518–7.891**	**0.003**	1.526	0.589–3.953	0.384
Previous VB		**0.212**	**0.088–0.51**	**0.001**	**0.310**	**0.126–0.762**	**0.011**
Previous BI		1.532	0.800–2.937	0.198			
CRP (≥ 10)		**2.922**	**1.500–5.693**	**0.002**	**2.192**	**1.086–4.427**	**0.029**
Anemia		1.432	0.743–2.762	0.284			
High Ferritin		**2.251**	**1.146–4.424**	**0.019**	1.016	0.436–2.371	0.970

Values in bold indicate statistically significant tests

*MELD* model for end stage liver disease,* HCC* hepatocellular carcinoma,* VB* variceal bleeding,* BI* bacterial infection,* CRP* C-reactive protein,* HR* hazard ratio,* CI* confidence interval

Multivariate Cox regression model revealed that high (Q4) ferritin level was an independent risk factor of 2-year mortality in the whole patient cohort (HR: 2.143, CI 1.174–3.910, *p* = 0.013) besides disease severity indicators and presence of HCC (Table 5A). However, when we split the cohort into compensated and decompensated patients, we observed this association only in the compensated cirrhosis group (HR: 4.367, CI 1.466–13.009, *p* = 0.008; Table 5B). In decompensated patients, clinical factors were found to be stronger predictors of mortality (Table 5C).

## Discussion

In a large cohort of cirrhotic outpatients, we measured serum ferritin levels and evaluated its association with the development of disease-specific complications and mortality during a 2-year-long follow-up period. We found that ferritin’s serum concentration was significantly higher in males and older patients, as it was previously reported [27]. We also observed significantly lower levels in patients, who previously had variceal bleeding while anemia was not associated with such difference in these patients. However, when we investigated correlations of ferritin with other laboratory measures, we found that parameters associated with hematopoiesis and iron homeostasis (hemoglobin, hematocrit, mean corpuscular hemoglobin and mean corpuscular volume) correlated with ferritin in both compensated and decompensated groups, while inflammation markers and disease severity measures did only in the latter group. This result indicates that although in advanced stages of cirrhosis, inflammation and disease severity are important modulators of ferritin level, in earlier stages, ferritin concentration mainly corresponds to the iron homeostasis of the body, much like in the healthy population. Previous studies reported that cirrhosis induced immune-dysfunction predominantly manifests in decompensated stage resulting in systemic inflammatory and compensatory anti-inflammatory responses [28]. This observation is in line with the lack of correlation between ferritin level and inflammatory measures in our compensated outpatients.

Aalan–Johansen and Kaplan–Meier analyses revealed that compensated patients with low serum ferritin levels had the highest incidence of decompensation during follow-up, while decompensated patients with high ferritin levels had the highest incidence of BI development. Mortality was associated with high ferritin level in every patient group. Previous studies reported association only between high ferritin levels and worse outcomes, however these investigations were conducted involving patients with ongoing AD [23–25]. Our observation that low serum ferritin concentration is associated with an increased risk of developing a decompensated clinical stage in patients with previously compensated cirrhosis is definitely an interesting and unexpected novel finding. Hence, some speculation was needed in order to interpret these results. Based on the observed correlations, we assume that compensated patients, who did not have previous acute bleeding but had low ferritin levels might have had chronic, subclinical (unrecognized) gastrointestinal hemorrhage leading to depleted iron stores and low ferritin concentration. In cirrhotic patients, this form of bleeding is mostly due to increased portal pressure (reaching the clinically significant threshold), which is a well characterized risk factor of AD [2]. When we checked gastroscopy results of patients who had low ferritin levels and developed decompensated clinical stage during follow-up, we found that 7 out of 8 patients had esophageal varices and/or portal hypertensive gastropathy at time of recruitment. This observation provides evidence for the presence of increased portal pressure in these patients. Consistently, in multivariate Cox regression, besides low ferritin level, anemia was also an independent predictor of the development of first AD events, further supporting the hypothesized connection between hidden blood loss and decompensation. However, the presence of chronic bleeding still needs to be verified by specific methods, for instance, fecal blood test in future studies. With that being said, ferritin measurement seems to have the potential to provide a very important additional screening tool in the evaluation of both early and advanced stage cirrhosis, aiding the appropriate clinical decisions in the future.

On the other hand, since low ferritin level was associated with higher incidence of decompensation, it might seem controversial that mortality was associated with higher ferritin levels in the compensated population. As discussed, elevated ferritin level indicates either iron overload, which is toxic to cells, or increased inflammation, caused by either bacterial translocation or a subclinical starting infection [16]. These last two, in return, can lead to overt infections, while inflammation in general and iron-induced toxicity can trigger the progression of HCC [29–31] and comorbidities [32–35] ultimately leading to increased overall mortality. Indeed, as Fig. 6 shows, almost every patient with high ferritin level who developed decompensated clinical stage died, and some deceased even without having an AD episode. In contrast, as Fig. 3 demonstrates, no patient with low ferritin level who did not develop decompensated clinical stage died during follow up. Furthermore, approximately 40% of those with low ferritin levels who did decompensate, also survived. The explanation for this difference is not clear, but the decompensation of non-inflammatory mechanisms, indicated by lower ferritin levels, might represent a less severe/more manageable type of AD.

However, uncovering all the underlying processes will require a complex perspective for future studies since ferritin is a multifunctional molecule that has the potential to even play a direct role in the development of various adverse events. As it has been reported, the two subunits serve different purposes: H-ferritin bears ferroxidase activity while acidic residues on L-ferritin facilitate the ferroxidase turnover and are essential for the nucleation of ferric (Fe^3+^) iron into the core of ferritin [16]. Importantly, H-ferritin’s cytosolic iron sequester activity has been shown to prevent iron-driven oxidative inhibition of glucose-6-phosphatase to sustain glucose production via liver gluconeogenesis, which ultimately increases the host’s tolerance to sepsis [12]. Another study showed that high level of L-ferritin inhibits LPS-mediated production of tumor necrosis factor-α and interleukin-1 in macrophages [36]. In contrast, a study investigating hematopoiesis in mice after the injection of human H-ferritin observed a myelosuppressive effect of the protein that depended on its ferroxidase activity and involved the induction of interleukin-10. Consistently, L-ferritin, lacking ferroxidase activity, did not display such an effect [37]. Since routine laboratory methods are designed to measure L-ferritin, we do not have information about the ratio of H-ferritin or iron saturation of serum ferritin, which could expand our understanding of the underlying processes. Additionally, transferrin level and saturation as well as liable iron level could have further supported our hypothesis about how low ferritin level is connected to decompensation. The lack of these data is a limitation of the present study and must be assessed by further investigations; however, the association of low ferritin level and low iron storage is evident even without the aforementioned laboratory results. Depleted iron stores may be treated using different iron salt supplementations. Recently, intravenous iron sucrose treatment has been reported to improve anemia specifically in patients with cirrhosis and gastrointestinal bleeding [38].

Finally, in decompensated patients, high ferritin level was associated with both investigated outcomes (BI and mortality). Yet, in this subpopulation, ferritin level lost statistical significance in the multivariate model of mortality (i.e., could not predict the outcome independently of other factors involved in the model). This observation may be explained by the detected correlations between ferritin level, inflammatory markers and disease severity scores in these patients, as similar correlations were not seen in the compensated subpopulation.

In conclusion, both low and high ferritin concentrations are more detrimental than normal levels, but for different hypothesized reasons. Both indicate distinct pathological processes playing roles in the development of different adverse outcomes. In cirrhotic patients who did not have previous acute bleeding but have low ferritin levels, the physician should consider looking for occult blood loss, while all patients with high ferritin levels are subject to increased mortality risk and require closer follow-up or intervention. These novel results need further exploration, but they are of importance and may support the need for including ferritin among markers routinely used to follow disease progression of patients with LC.

## Data Availability

The datasets generated and/or analyzed during the current study are not publicly available due to national regulations concerning patients’ data handling (third parties cannot inspect clinical data stored for research purposes without recording their name, reason and time of access) but are available from the corresponding author on reasonable request.

## Abbreviations

AD: Acute decompensation
ACLF: Acute-on-chronic liver failure
BI: Bacterial infection
CI: Confidence interval
CRP: C-reactive protein
HE: Hepatic encephalopathy
HR: Hazard ratio
INR: International normalized ratio
IQR: Interquartile range
LC: Liver cirrhosis
MELD: Model for end stage liver disease
sHR: Sub-distribution hazard ratio
